# Dressler Syndrome: Not Just a Relic of the Past

**DOI:** 10.7759/cureus.30670

**Published:** 2022-10-25

**Authors:** Kristopher Aten, Kenneth Raney, Anas Alomar

**Affiliations:** 1 Internal Medicine, Methodist Health System, Dallas, USA; 2 Interventional Cardiology, Methodist Southlake Hospital, Southlake, USA

**Keywords:** pericardial effusion, myocardial infarction, cardiology, post-mi syndrome, pericarditis, dressler syndrome

## Abstract

Dressler syndrome, also known as post-myocardial infarction syndrome, is a condition in which sterile pericarditis develops shortly after myocardial injury. It is characterized by pericardial inflammation typically including pericardial effusion, pleuritic chest pain, and elevated inflammatory markers. While its incidence has greatly decreased in the modern era of coronary revascularization, it remains a clinically important entity with the potential for serious morbidity. Here we present a case of presumed Dressler syndrome in a 54-year-old male who presented to the emergency room with a recurrence of chest pain and clinical signs of pericarditis after sustaining an anterior myocardial infarction two weeks previously.

## Introduction

Post-myocardial infarction syndrome (post-MI syndrome), also known as Dressler syndrome, is a clinical condition characterized by sterile pericarditis that develops after a known cardiac injury [1]. Dressler syndrome is commonly associated with acute myocardial infarction (MI) but can present after cardiac surgeries, trauma to the thorax, electrophysiology (EP) procedures, and other insults to the myocardium or surrounding tissues. While several pathologic mechanisms have been proposed, there is consensus that this syndrome develops at least in part via activation of the immune system in response to myocardial damage leading to immunologic cross-reactivity and pericarditis. It is felt that tissue infarction causes release of intracellular components leading to auto-antibody production against myocardial and pericardial tissue [2]. Post-MI syndrome classically presents with typical signs and symptoms of pericarditis (pleuritic chest pain, pericardial effusion, ECG with diffuse ST elevation and PR depression) which develop within one week of MI [3]. While historically more common, the improvement in coronary revascularization technologies has made Dressler syndrome a relatively rare occurrence with an estimated incidence of less than 1% of post-MI patients [4-5].

## Case presentation

A 54-year-old male with a history of uncontrolled diabetes, hypertension, tobacco use, and a cardiac history as outlined below presented to the emergency room with acute onset left-sided chest pain that awoke him from sleep. The pain was described as sharp, constant, and accompanied by dyspnea and radiation to his left arm. Self-administration of nitroglycerin prior to arrival did not relieve his pain. Review of his recent medical records revealed that the patient presented to an out-of-state emergency department two weeks prior complaining of crushing, sub-sternal chest pain. At this outside facility, he suffered two episodes of cardiac arrest (ventricular fibrillation) with successful resuscitation. He was transferred to a higher level of care, where he underwent left heart catheterization (LHC) and placement of two stents in the left anterior descending artery (LAD) with subsequent discharge home. The level of stenosis was not specified in his limited records. Over the next several days, he experienced multiple episodes of recurrent chest pain prompting two repeat hospitalizations and two additional LHCs. Both catheterizations revealed depressed but stable left ventricular ejection fraction (LVEF) of ~40%, patent LAD stents, and previously known 50% stenosis of his right coronary artery. He was discharged after brief observation both times.

**Figure 1:**
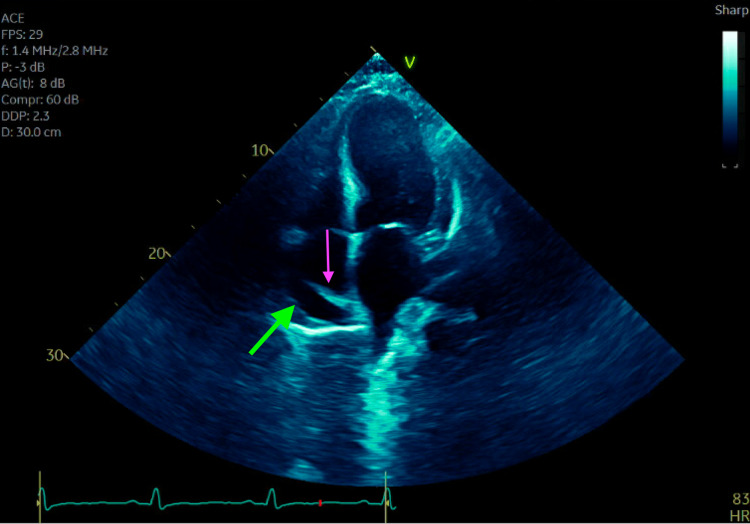
Trans-thoracic Echocardiogram (TTE) View of Pericardial Effusion Still image of the apical four-chamber view of the patient's transthoracic echocardiogram demonstrating a small pericardial effusion (green arrow) and diastolic collapse of the right atrium (pink arrow).

On presentation to our emergency department, he was noted to be hypertensive with a blood pressure of 184/91, a pulse of 93bpm, and otherwise normal vitals. Electrocardiogram was notable for diffuse t-wave flattening but no ST elevation or other acute ischemic changes. His initial troponin I level was elevated at 0.181ng/mL but considerably lower than his last available value of 3.66ng/mL noted 13 days earlier on discharge from the previous hospital. Given the relative decrease in his troponin I measurement, this elevation was deemed to represent resolving myocardial injury from his recent MI rather than ongoing ischemia. The remainder of his laboratory workup, including complete blood count and comprehensive metabolic panel, was remarkable only for hyperglycemia and mild normocytic anemia with a hemoglobin of 13.2g/dL. Given the patient’s recent and extensive cardiac history, cardiology was consulted early, and the patient was admitted for further monitoring and workup. Trans-thoracic echocardiogram (TTE) was obtained and revealed an LVEF of 35-40% and severe hypokinesis of the anterior wall, both consistent with his initial post-arrest TTE, as well as a small new pericardial effusion (Figure 1). Given the delay between MI and recurrence of chest pain, the nature of his pain, and workup showing a pericardial effusion without signs of in-stent thrombosis or other new ischemic pathology, the patient was presumptively diagnosed with Dressler syndrome. Because his symptoms improved over the next 24-48 hours with only a very small pericardial effusion, treatment with colchicine and high-dose non-steroidal anti-inflammatory drugs (NSAIDs) was deferred. The patient was monitored for another day and discharged home with improvement in his symptoms and stable vital signs and lab values.

## Discussion

Post-MI syndrome has always been a relatively rare complication of MI. Estimates vary but even prior to the advent of coronary revascularization, its incidence was estimated to be in the range of 3-5% by some studies. With the emphasis on rapid revascularization as the cornerstone of treatment in acute MI, the incidence of post-MI syndrome has dropped even further and is now estimated to develop in as few as 1% of patients after MI [4-5]. As described above, the triad of pleuritic chest pain, pericardial effusion, and ECG findings of diffuse ST elevation and PR depression in a patient post-MI are classic for the diagnosis of post-MI syndrome. Importantly for clinicians, this triad has very poor sensitivity and is present in only a minority of patients. One study of post-MI syndrome patients found that classic ECG changes were present in only ~24% of cases and another found pericardial effusion to be absent in nearly 20% [6]. While typically not life-threatening, serious complications such as cardiac tamponade have been described [7]. Pain and fatigue can also have significant impacts on the quality of life for patients with persistent or recurrent symptoms, justifying treatment with colchicine and NSAIDs in most cases [8-9].

## Conclusions

The case above demonstrates the risk of underdiagnosis when relying on the classic triad to diagnose post-MI syndrome. While the patient described above was found to have a pericardial effusion which strengthened clinical suspicion for post-MI syndrome, he presented with atypical pain for pericarditis without the expected ECG findings. These atypical cases, while more common than the classic triad, often lead to delayed or incorrect diagnoses. To increase prompt diagnoses of post-MI syndrome, clinicians should maintain a degree of suspicion for any patient presenting with new chest pain, fatigue, or signs/symptoms of active inflammation after sustaining MI. Additional lab tests to evaluate inflammation such as the erythrocyte sedimentation rate or c-reactive protein can play an important role in securing a diagnosis. TTE is also recommended to evaluate for pericardial effusion and to rule out any objective evidence for hemodynamically significant effusion or other structural pathology. Once post-MI syndrome is confirmed or strongly suspected, treatment with colchicine and high-dose non-steroidal anti-inflammatory agents such as aspirin or indomethacin should be considered to prevent refractory symptoms.
